# Supplementary material for: Erythrocytes retain hypoxic adenosine response for faster acclimatization upon re-ascent
Source: Nat Commun. 2017 Feb 7;8:14108. doi: 10.1038/ncomms14108 (PMC5309698; doi:10.1038/ncomms14108)
Supplement: Supplementary Information — Supplementary Figures [file ncomms14108-s1.pdf]

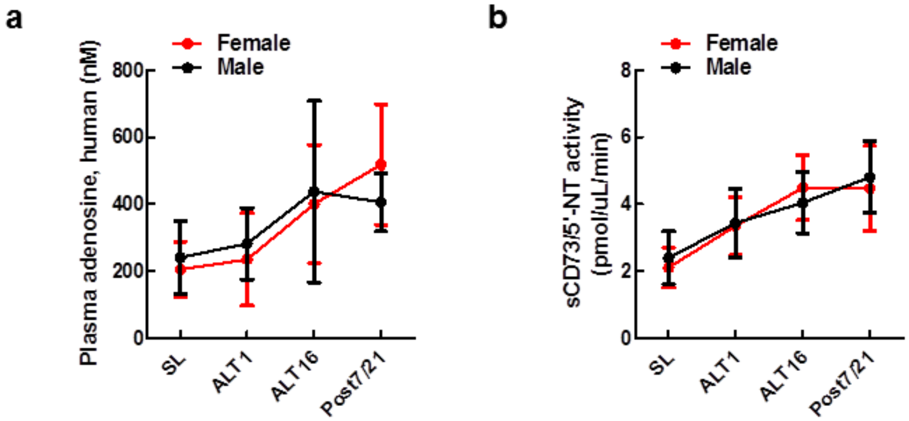

Supplementary Fig. 1. The effect of genders in sCD73/5'-NT activity and plasma adenosine during high altitude hypoxia. (a) There are no differences in plasma adenosine levels at each time point between female and male volunteers (female volunteers=8, male volunteers=9). (b) There are no differences in sCD73 enzyme activities at each time point between female and male volunteers (female volunteers=9, male volunteers=11).

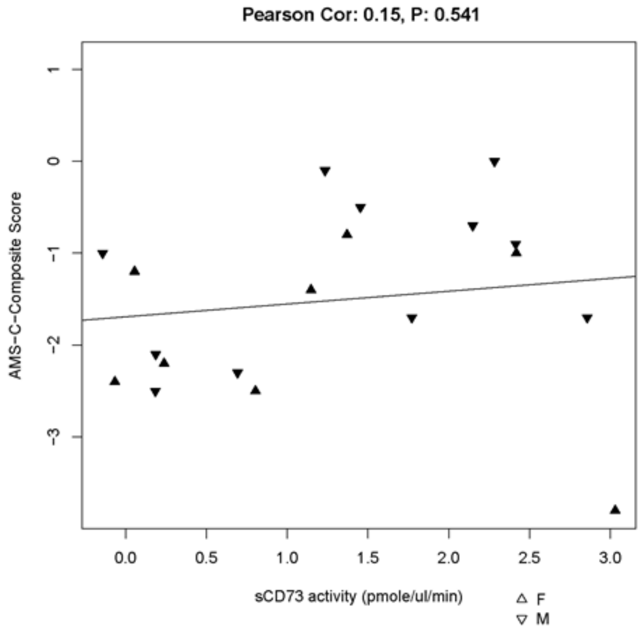

Supplementary Fig. 2. The correlation between sCD73/5'-NT activity and AMS-C composite score. There are no significant correlation between sCD73-NT activity and AMS-C composite score (Pearson correlation, n=21, *P=0.541*).

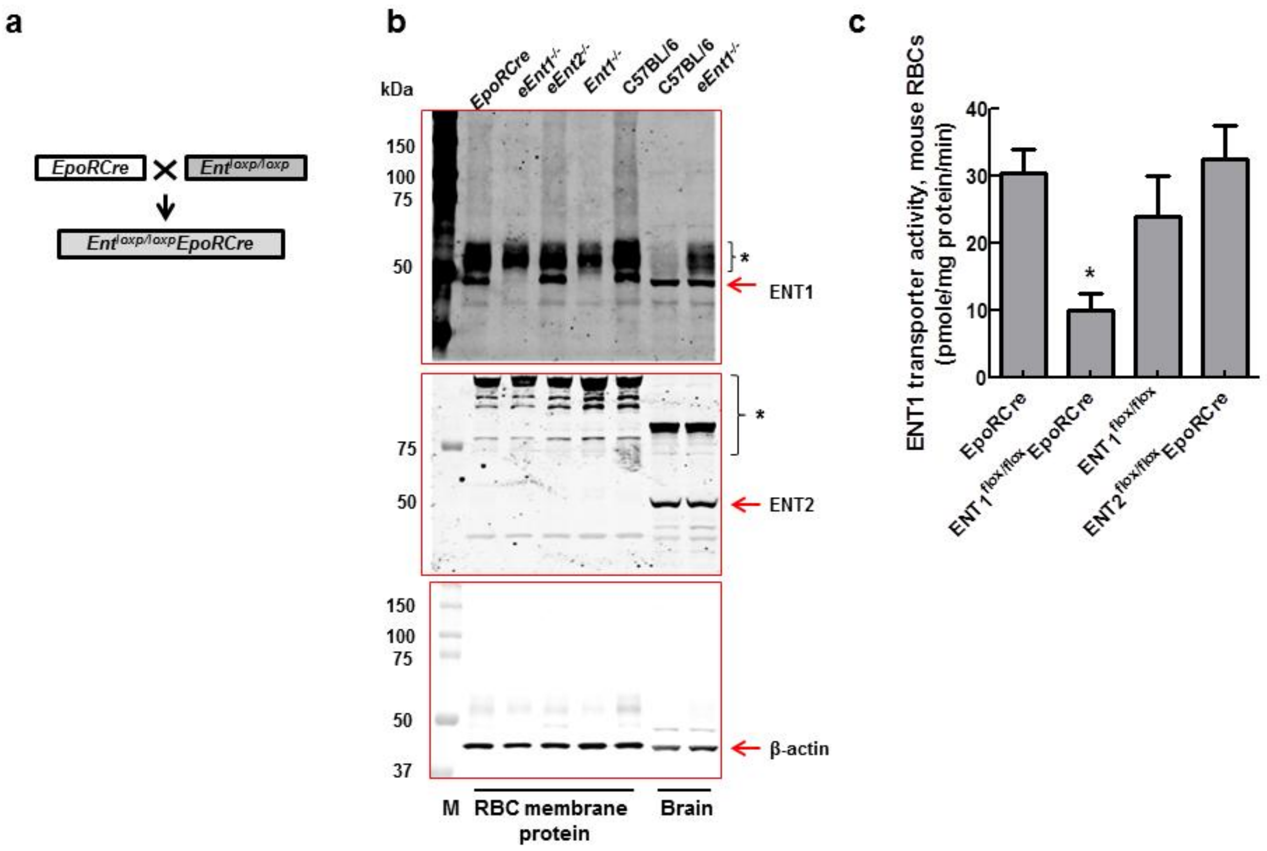

Supplementary Fig. 3. The role of eENT1 in the uptake of extracellular adenosine. (a) Illustration of generation of eENT1 mutant. (b) eENT1 is dramatically lost in erythrocyte, but not other tissue in *Ent1*$^{flox/flox}$*/EpoRCre* mice, *: non-specific band (primary antibody used in this western blot is mouse mono-clonal antibody), M: protein marker. (c) eENT1 activity in RBCs from erythrocyte-ENT knockouts judged by *in vivo* adenosine uptake assay (n⩾4, mean±SD, *P<0.05, t test*).

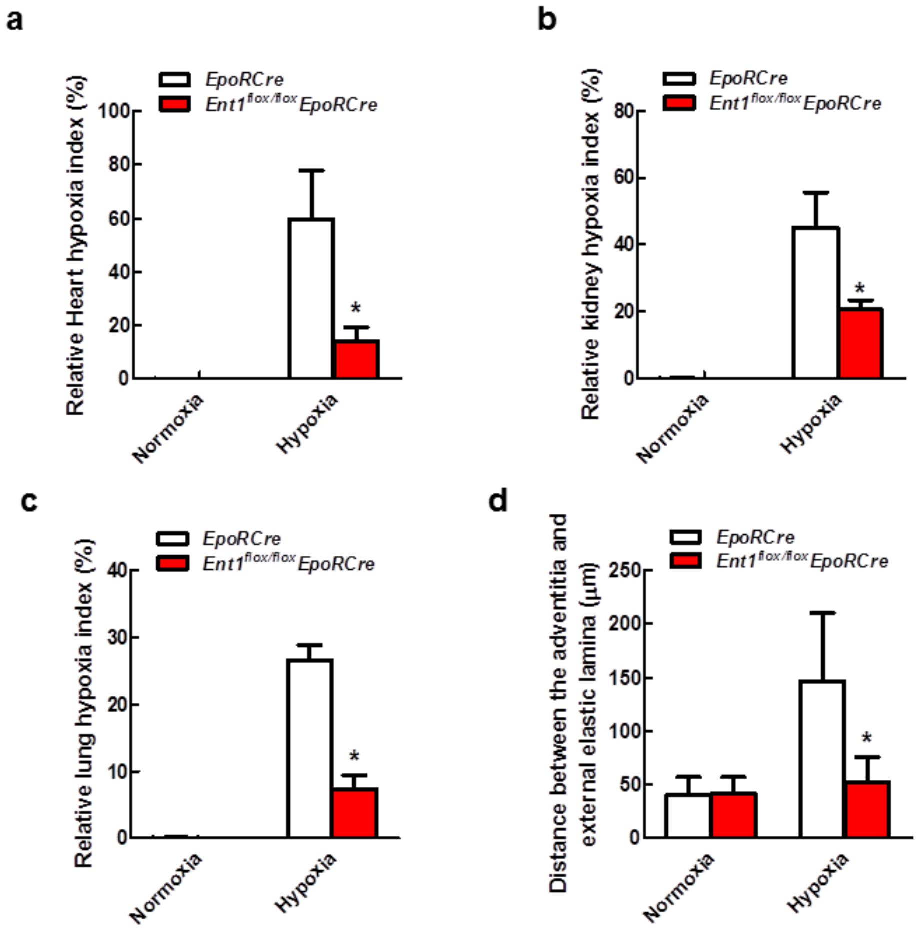

Supplementary Fig. 4. (Related to Fig. 2) Reduced tissue hypoxia was observed in eENT1 knockout (*Ent1*$^{flox/flox}$*/EpoRCre*) mice compare to control mice (*EpoRCre*) after 72 hours hypoxia treatment by Hypoxia probe. (a) Statistical results of Fig. 2e, heart (n=5, mean±SD, *P<0.05, t test*). (b) Statistical results of Fig. 2e, kidney (n=5, mean±SD, *P<0.05, t test*). (c) Statistical results of Fig. 2e, lung (n=5, mean±SD, *P<0.05, t test*). (d) H&E stained lung section from 72 hours-hypoxia treated animals revealed perivascular fluid accumulation, statistical results (n⩾35 views of edema from animals n⩾4, mean±SD, *P<0.001, t test*).

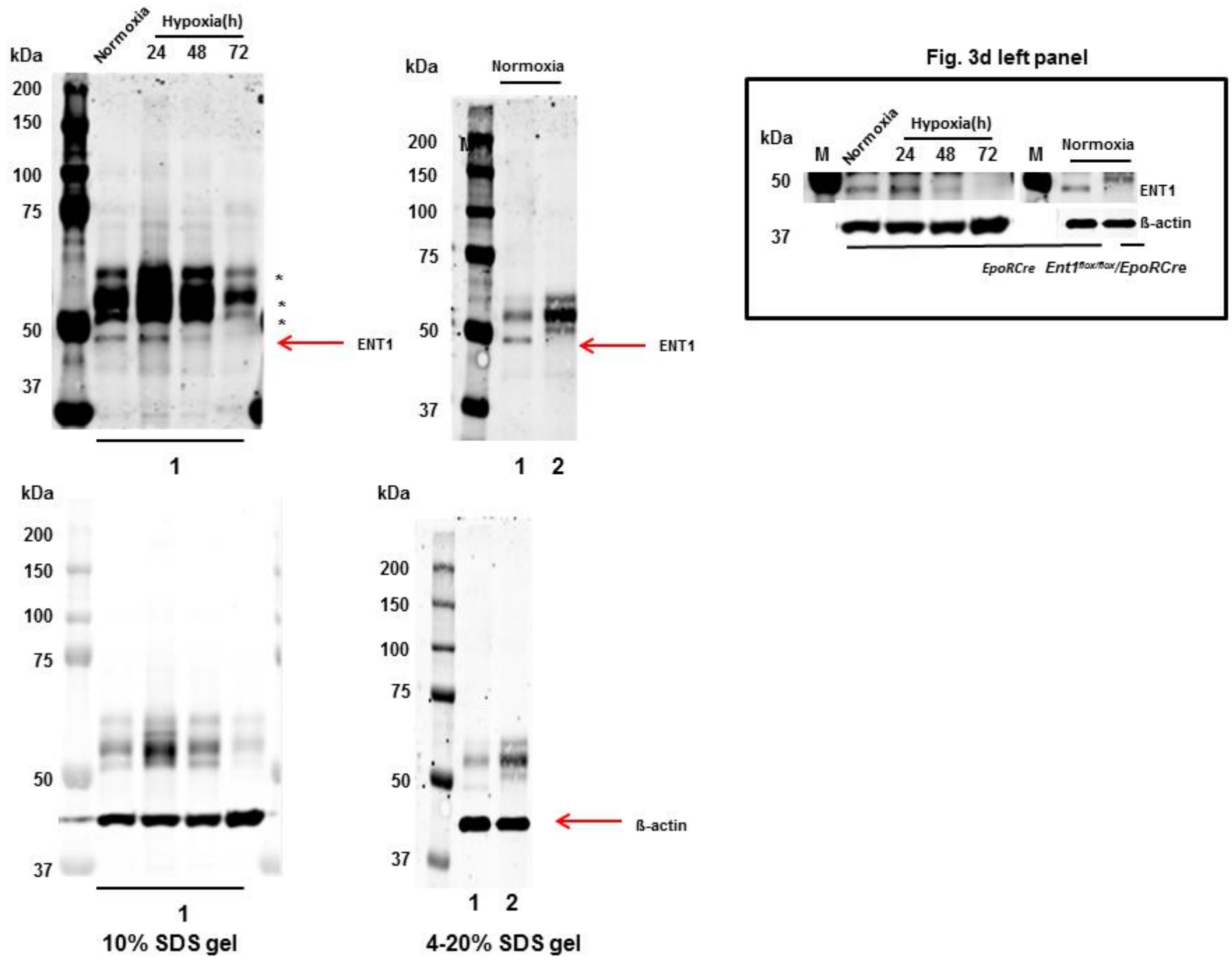

Supplementary Fig. 5. Related to Fig 3d left panel, western blot image, 1: *EpoRCre***,** 2: *Ent1*$^{flox/flox}$*/EpoRCre*, *: non-specific bands.

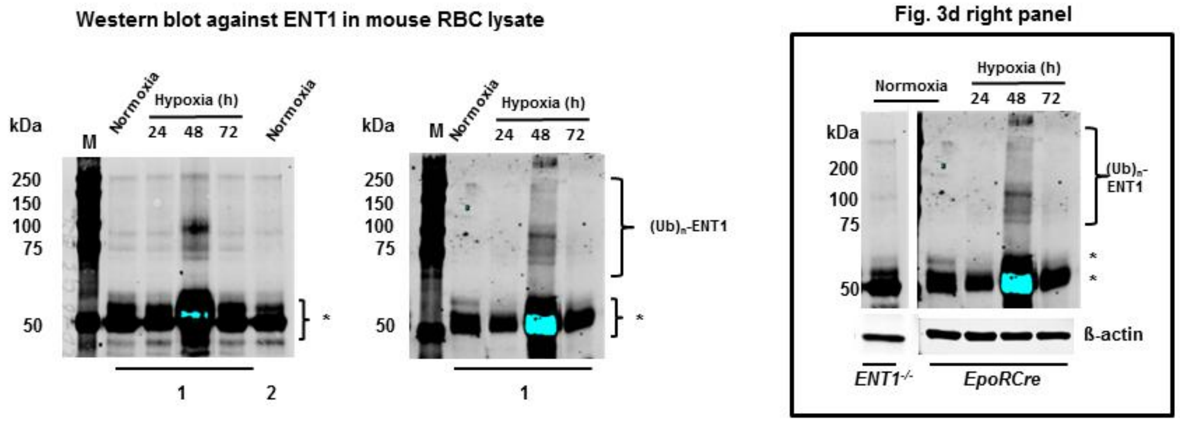

Supplementary Fig. 6. Related to Fig. 3d right panel, western blot image. 1: *EpoRCre*, 2: *Ent1*$^{-/-}$, *: non-specific bands.

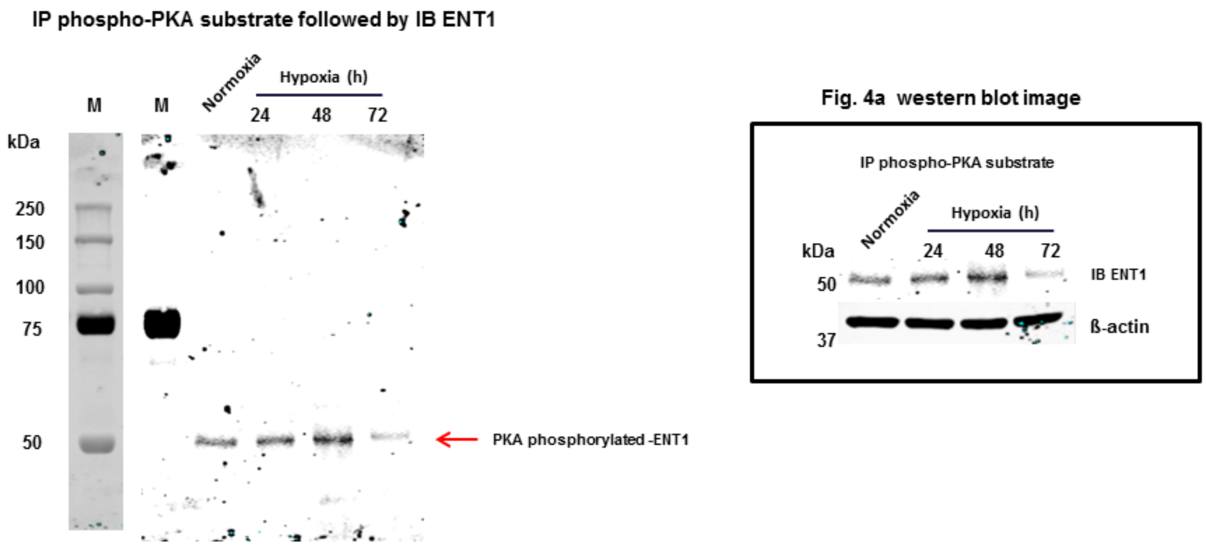

Supplementary Fig. 7. Related to Fig. 4a, western blot image, M: protein maker.

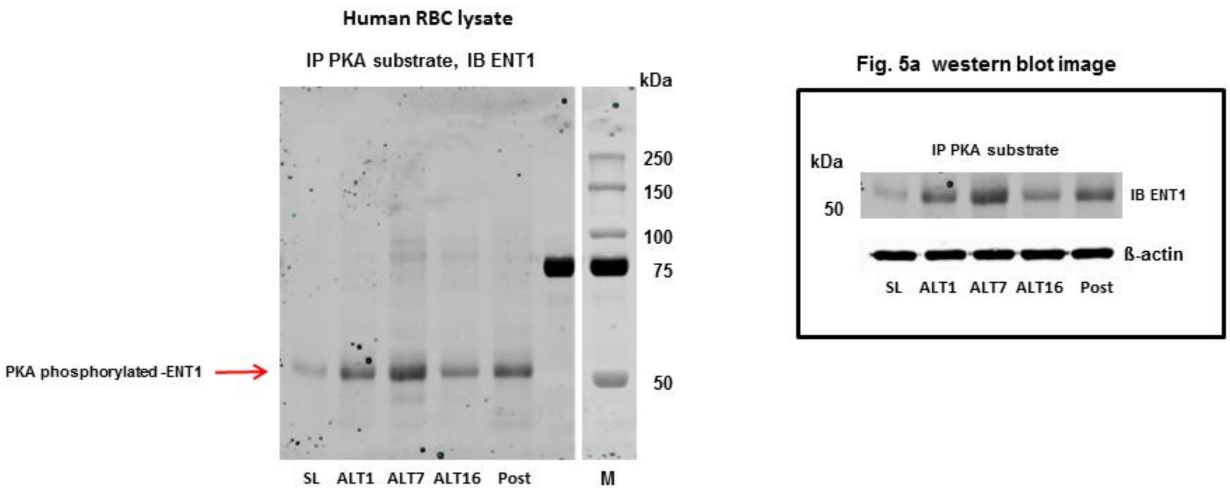

Supplementary Fig. 8. Related to Fig. 5a, western blot image, M: protein maker.

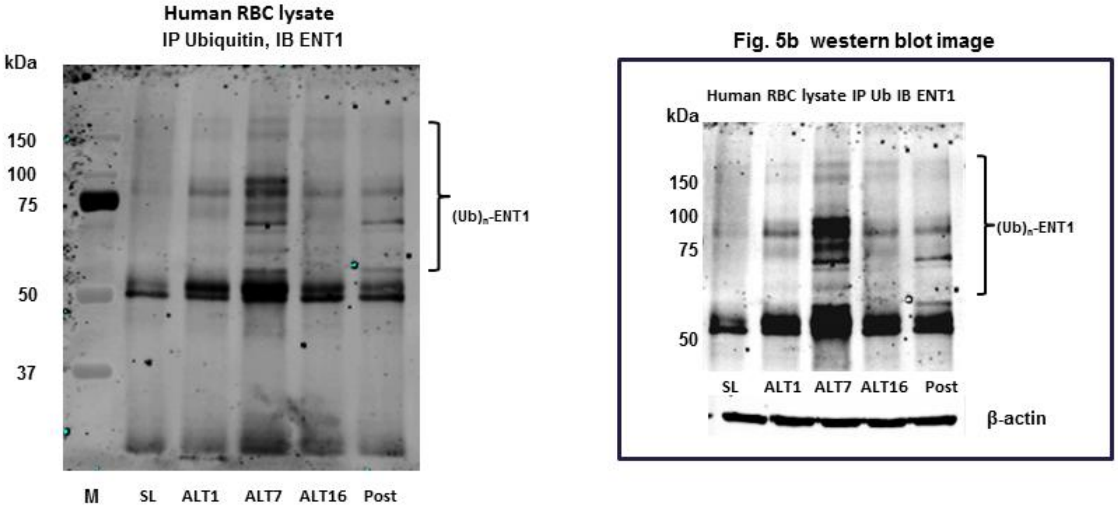

Supplementary Fig. 9. Related to Fig. 5b, western blot image, M: protein maker.

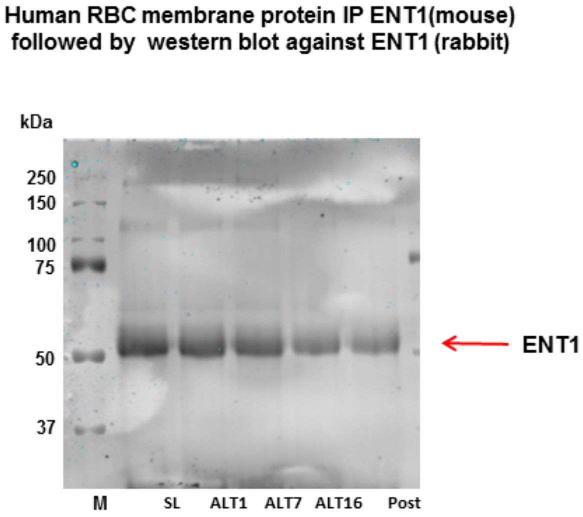

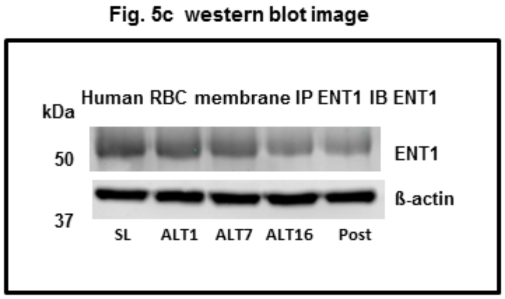

Supplementary Fig. 10. Related to Fig. 5c, western blot image, M: protein maker.
